# Unravelling the unsolved paradoxes of cytokine families in host resistance and susceptibility to *Leishmania* infection

**DOI:** 10.1016/j.cytox.2020.100043

**Published:** 2020-10-12

**Authors:** Bernard Ong'ondo Osero, Raphael Taiwo Aruleba, Frank Brombacher, Ramona Hurdayal

**Affiliations:** a Division of Immunology, Department of Pathology, Faculty of Health Sciences, Institute of Infectious Diseases and Molecular Medicine (IDM), South African Medical Research Council (SAMRC) on Immunology of Infectious Diseases, University of Cape Town, Observatory 7925, Cape Town, South Africa; b International Centre for Genetic Engineering and Biotechnology, Cape Town Component, Observatory 7925, Cape Town, South Africa; c Department of Molecular and Cell Biology, University of Cape Town, Rondebosch 7701, Cape Town, South Africa; d Faculty of Health Sciences, Wellcome Centre for Infectious Diseases Research in Africa, Institute of Infectious Diseases and Molecular Medicine (IDM), University of Cape Town, Observatory 7925, Cape Town, South Africa

**Keywords:** Leishmaniasis, Cytokine families, Disease-enhancers, Host-protectors

## Abstract

Leishmaniasis is a neglected disease caused by protozoan parasites of the genus *Leishmania*. Successful clearance of *Leishmania* relies on a robust human immune response and various cytokines have been implicated in resistance and susceptibility to *Leishmania* infection. Accordingly, various immunotherapeutic approaches involving cytokines and cytokine receptors are being considered as novel avenues of treatment given the limited efficacy of current anti-leishmanial drugs. These approaches target canonical T helper (Th)1/Type 1 cytokines as intended mediators of host-protection to infection whilst concomitantly suppressing Th2/Type 2 cytokines and their anticipated disease-promoting roles. However, the use of cytokine and cytokine receptor gene-deficient mice over the years has challenged this simplistic view of Th1/Type 1-mediated resistance and Th2/Type 2-mediated susceptibility. Indeed, contribution to susceptibility vs resistance is only a partial consequence to cytokine action as the overall response is multi-faceted due to the pleiotropic, redundant, antagonistic and synergistic action of cytokines and interactions with immune cells in the diseased state. Notably, while the responses of certain cytokines are selectively host-protective or characteristic disease-enhancers, some ligands exert a response depending on the parasite-species initiating infection. Paradoxically, others play dual or contradictory roles in different *Leishmania* immunopathologies. Hence, cytokines in disease is an unsolved paradox and a comprehensive knowledge of cytokine interplay is important to guide the development of novel immunotherapeutics against leishmaniasis. In this review, we characterize various cytokine families in persistence and clearance of the *Leishmania* parasite and particularly elucidate unsolved cytokine puzzles in leishmaniasis based on information acquired from “gain of knowledge by loss of function” studies in cytokine and cytokine receptor gene-deficient mice.

## Highlights

• Cytokine communication in leishmaniasis begets host-resistance or susceptibility.
• All cytokine families, except IFN, overlap as host-protectors or disease-enhancers.
• IFN-γ and IL-12 are unequivocally anti-leishmanial.
• IL-21, TGF-β and IL-10 are unequivocally disease-enhancing in VL.
• Cutaneous leishmaniasis has the most unsolved cytokine paradoxes.

## 1 Introduction

Leishmaniasis is a neglected tropical disease caused by over 20 species of obligate intracellular protozoan parasites called *Leishmania* and are transmitted by the bite of female sandflies [1]. In humans, severity of pathology depends on the infecting spp. and the major clinical manifestations are cutaneous leishmaniasis (CL), mucocutaneous leishmaniasis (MCL) and visceral leishmaniasis (VL). Worldwide, this disease is reported as a public health burden in 102 countries with approximately one million new cases annually and one billion people residing in these regions at risk of infection [2]. Control of this infection relies exclusively on anti-leishmanial drugs including pentavalent antimonials (SbV), amphotericin B, miltefosine, and paromomycin [3]. However, the rise of drug resistance has limited their efficacy coupled with misuse and drug toxicity [4]. Considering there is no effective vaccine, it is imperative that the efficacy of these drugs be enhanced to expand their lifespan in clinical use. One approach gaining momentum is immunotherapy either alone, or co-administration with a drug in an immunochemotherapeutic approach.

**Fig. 1:**
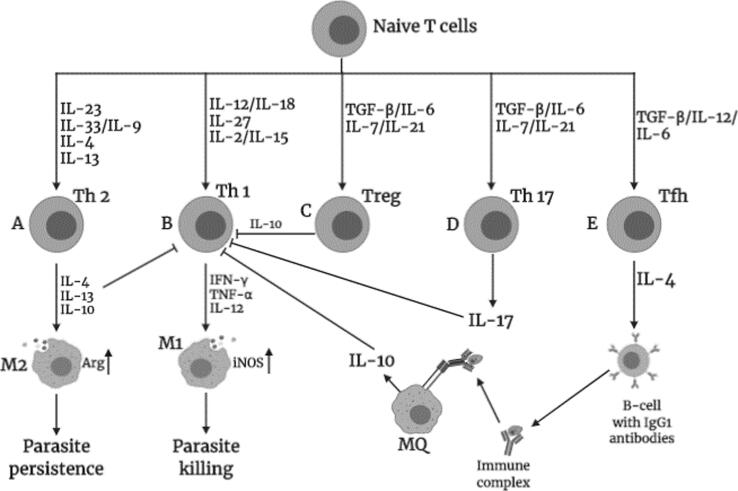
**Potential events that could be triggered by various cytokines on naïve T cells leading to polarization of different T helper (Th) subsets associated with healing and non-healing responses during *Leishmania* infection in mice**. **A**) Th2 expansion, mediated by IL-23, IL-9 together with IL-33 or IL-4 and IL-13, leads to the expression of canonical Th2 cytokines (IL-4, IL-13 or IL-10) that trigger arginase-induced alternative macrophage (M2) activation, which promotes parasite persistence. **B**) Differentiation of naïve T cells into Th1 can be induced by IL-12 and IL-18, IL-27 or via combination of IL-2 with IL-15. IFN-γ released by Th1 cells classically activates macrophages (M1) via inducible nitric oxide synthase (iNOS) to release nitric oxide (NO) that kills intracellular parasites. **C**) IL-6, with help of TGF-β or IL-27/IL-21, can polarize naïve cells to T regulatory cells (Treg) that produce IL-10, which immunosuppresses Th1 effects via induction of IL-10. **D**) TGF-β, with help of IL-21, influences expansion of Th17 cells that can suppress Th1 immune responses. **E**) Naïve T cells can differentiate into T follicular helper (Tfh) cells by TGF-β, IL-12 or IL-6 that produce IL-4 which induces class switch of B cells into IgG1-secreting plasma cells. Antibodies opsonize parasites, which enhances phagocytosis but stimulates macrophages to produce IL-10 that suppress Th1 effects. Figure created in BioRender.com.

**Fig. 2:**
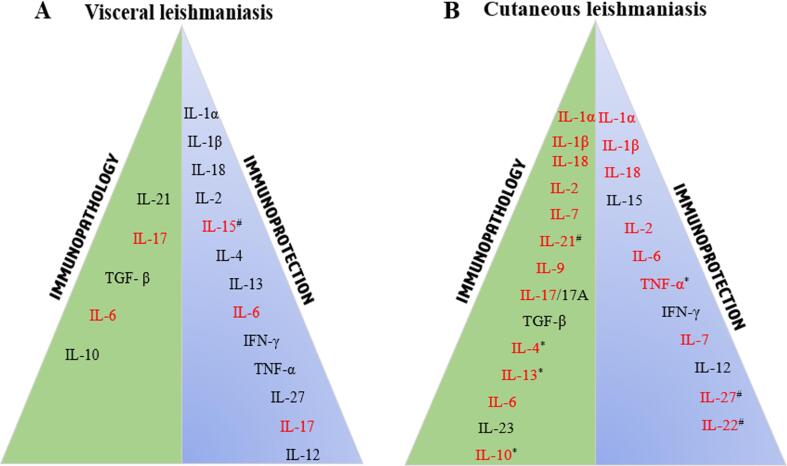
**Cytokines involved in immunopathology and immunoprotection** in (**A**) visceral leishmaniasis (VL) and (**B**) cutaneous leishmaniasis (CL). The green right triangle shows disease-enhancing cytokines that trigger immunopathology in CL and VL, associated with parasite persistence and non-healing disease. The blue left triangle depicts cytokines involved in host-protection against VL and CL that is associated with parasite clearance and healed phenotype. However, cytokines in red appear to play a paradoxical role in both host protection and susceptibility. Noteworthy, only IFN-γ and IL-12 are unequivocally host-protective in both CL and VL. In CL, more cytokines remain unsolved paradoxes than in VL. *denotes species-specific paradox; ^#^denotes paradox due to no changes in protection/susceptibility.

Caution however must be noted as infection involves a complex interplay between host and parasite. Nonetheless, macrophage activation via IFN-γ is critical to kill parasites (Fig. 1). Accordingly, earlier studies documented that T helper (Th) 1 and Th2 CD4^+^ T-cell populations control resistance and susceptibility to infection, respectively, which was extrapolated to immunotherapies and vaccination but often did not reach the desired effects, highlighting that there are many complexities in immunity against leishmaniasis (Fig. 1) [5]. It is therefore not surprising that with the introduction of cytokine/cytokine-receptor gene-deficient mouse models, certain aspects of this dichotomy have been extensively challenged. For instance, IL-4 and IL-13 are canonical Th2 cytokines noted to exacerbate CL yet the same cytokines are host-protective against VL by instructing Th1 immune responses [6]. In parallel, as defined in this review, some cytokines exert a dual role as a protective and progressive factor in CL or VL pathology, ultimately classifying it as an unsolved paradox (Fig. 2). Towards providing a rational framework for consideration in the design of novel immune-based therapies, this review outlines and discusses the solved and unsolved paradoxes of various cytokine families (Fig. 2) based on information gleaned from gene-deficient murine models of leishmaniasis.

## 2 Interleukin 1 family of cytokines

The interleukin (IL)-1 family is a group of 11 cytokines and 10 receptors. Typically, 7 cytokines exhibit agonistic activity (IL-1α, IL-1β, IL-18, IL-33, IL-36α, IL-36β and IL-36γ) and the remaining four ligands exhibit antagonistic functions (IL-1 receptor antagonist (IL-1Ra), IL-36Ra, IL-37 and IL-38). Members of the IL-1 family polarize T-cell functions, promotes innate immunity and assists in the maintenance of inflammatory processes [7].

### 2.1 IL-1α and IL-1β are protective in VL but play a paradoxical role in CL

IL-1 is synthesized as a pro-protein and proteolytic cleavage leads to two mature forms, IL-1α and IL-1β. Although agonistic in theory, both proteins exhibit diverse responses to leishmaniasis. In B10.D2/n mice chronically infected with *L. donovani*, administration of recombinant IL-1α increased IFN-γ and granuloma production with minimal effect on the course of chronic infection [8]. This suggests that while IL-1α may synergize to increase IFN-γ, this alone is insufficient for inducing anti-leishmanial activity. Treatment of *L. major*-infected mice with IL-1α during T-cell priming mediates IL-12-induced Th1 induction [9]. Altogether, these studies highlight IL-1α as a potent inducer of Th1 immunity. Paradoxically, IL-1α has been postulated as a disease enhancer as IL-1α^-/-^ mice were more resistant to *L. major* infection than IL-1α-sufficient BALB/c mice [10]. In line with this, C57BL/6 IL-1RI^−/−^ mice treated with 50 ng IL-1α had increased lesions compared to WT mice [11]. Holistically, regulation of IL-1α during infection is an unsolved paradox as responses vary from protective to progressive, independent of *Leishmania spp*. Similarly, IL-1β plays a paradoxical role in CL but protective in VL.

The Nod-like receptor protein 3 (NLRP3) inflammasome pathway has been shown to be the main route for IL-1β production. Together, it restricts parasite replication during CL, MCL and VL, as demonstrated in inflammasome-deficient mice [12]. Mechanistically, this was due to IL-1β-mediated NO production via signaling through IL-1R and MyD88 [12] and dependent on the P2X7 receptor as demonstrated in P2X7-deficint C57BL/6 mice infected with *L. amazonensis* [13]. In contrast to a host-protective role, inflammasome-dependent IL-1β also shows disease-enhancing properties as C57BL/6 mice deficient for IL-1β cleared *L. major* infection [14]. Analogous to this, IL-1β treatment enhances disease pathology in BALB/c and C57BL/6 mice during *L. major* infection [15]. In comparison, IL-1α^-/-^ are slightly more resistant to *L. major* infection than IL-1β-knockout mice [10]. Hierarchically, this could suggest that IL-1α tips susceptibility more than IL-1β. During VL however, it is hypothesized that NLRP3 inflammasome-driven IL‐1β production is impaired via inhibition of NF‐κB activation [16], suggesting a possible role for IL-1β in host protection against *L. donovani*. Therefore, selective manipulation of IL-1β in the host as a host-directed therapy (HDT) has the potential to alleviate VL, however, more studies are needed to ascertain this observation.

### 2.2 IL-18 protects in VL but pathogenic in CL, especially in the absence of IL-12

IL-18 is a proinflammatory cytokine secreted by activated macrophages, monocytes and dendritic cells (DCs) and stimulates the development of Th1 and NK cells by concomitant inhibition of Th2 via IL-12. However, over-expression of IL-18 exacerbates inflammation, suggesting a delicate pathophysiological role for IL-18 [17], which has been documented in leishmaniasis. IL-18 is a susceptibility factor in CL since IL-18^-/-^ mice control infection compared to WT C57BL/6 mice [18]. Additionally, *L. major*-infected BALB/c mice treated with IL-18 showed enhanced lesions and higher Th2 responses compared to PBS-treated controls [19]. Paradoxically, IL-18-deficient C57BL/6 mice showed prolonged footpad swelling and reduced NO production [20]. Interestingly, IL-18^-/-^ mice were not deficient for Th1 cells as the administration of IL-18 rescued their phenotype suggesting that IL-18 synergizes with Th1 cells to secrete IFN-γ. Notably, co-administration of IL-12 and IL-18 in the first week after infection in BALB/c mice strongly activated Th1 cells and protective immunity [20] suggesting a synergistic mechanism for induction of Th1. Interestingly, while IL-18 and IL-12 are synergistic for Th1 in *L. major* and IL-18 plays a dual role, it protects from *L. donovani* infection and functions independently of IL-12 [21]. Hence, IL-18 and IL-12 responsiveness also vary by parasite strain [9], [22]. Overall, IL-18 appears to mediate Th1 immunity while it can also enhance Th2 responses in the absence of IL-12.

## 3 IL-2 cytokine family

This family of cytokines share a common receptor γ chain and members include IL-2, IL-4, IL-13, IL-7, IL-9, IL-15 and IL-21, that collectively regulate lymphocyte development, survival, growth and differentiation. In leishmaniasis, all members have received considerable attention.

### 3.1 IL-15 is a paradox but can synergise with IL-12 for anti-leishmanial activity, and IL-2 is anti-leishmanial in VL

IL-15 is secreted mainly by activated monocytes and like IL-2, signals via a trimeric receptor. Thus IL-15 exhibits immunological functions comparable to IL-2 such as T-cell proliferation and inhibition of apoptosis and B-cell maturation. Notably, IL-15 enhances the phagocytic ability of neutrophils and induces the activity of IFN-γ/IL-12 thereby regulating a Th1 phenotype [23], [24]. In leishmaniasis, IL-15 is controversial but IL-2 promotes anti-leishmanial responses.

In support, IL-15 stimulated killing of *L. infantum* in macrophages via secretion of IL-12 [25], highlighting that IL-15, like IFN-γ, is an inducer of IL-12. Indeed, *Leishmania* antigen-stimulated PBMC from CL patients incubated with anti-IL-2 and anti-IL-15 have decreased IFN-γ suggesting that IL-15 may enhance IFN-γ for protection [26]. Surprisingly, another study ruled out any protective role for IL-15 as endogenous IL-15 was dispensable for macrophage activation and Th1 development in acute VL [27]. Despite this, IL-12/15 stimulated PBMCs from *Leishmania infantum*-infected dogs alleviate immune cell suppression [28], suggesting that the combination of IL-15 with IFN-γ, IL-12 and/or IL-2 may boost anti-leishmanial Th1-mediated efficacy of IL-15. Thus, while IL-15 remains an unsolved paradox in VL infection, studies in CL seem to indicate protection but gene-deficient studies are under-explored.

Interleukin-2, like IL-15, is somewhat of a paradox. While it was initially reported as a susceptibility factor in CL via induction of IL-4 [29], it is also anti-leishmanial in VL patients via regulation of CD4^+^ T cells [30]. In CL patients, IL-2 and IL-15 are attributed to IFN-γ secretion, but in MCL patients, IL-2 and not IL-15 is needed for IFN-γ [26], highlighting non-redundant parasite-specific functions. In VL, IL-2 acts independently of IFN-γ to exert leishmanicidal activity [31]. Overall, IL-2 appears to be important in protection against VL but in CL, it is an unsolved paradox as it either enhances disease or protects against it. Available data does appear to implicate IL-15 in protection in synergy with IL-12, IFN-γ or IL-2.

### 3.2 IL-7 is under-explored in VL and an unsolved paradox in CL

The anti-microbial activity of IL-7 against *L. major* parasites has been demonstrated in macrophages and clearance reached 99% upon simultaneous treatment with IFN-γ highlighting synergistic roles for IL-7 and IFN-γ in the anti-leishmanial response [32]. This appears to be downstream of IL-7R as IL-7R expression during *L. major* infection contributed to the maintenance of Th1 effector cells [33]. In contrast to this, *L. major* infected BALB/c mice treated with IL-7 have enhanced lesion development characterized by higher amounts of IL-4 and IL-10 and reduced IFN-γ [34]. This increase in susceptibility was attributed to increased B lymphopoiesis and could be explained by B effector 2 B-cells mediating Th2 cell development and susceptibility to CL [35]. Interestingly, IL-7/IL-7R gene-deficient mice have not been utilised which could reconcile these observations. No studies of IL-7 during VL are available to-date.

### 3.3 IL-21 is a paradox but tips the scales as a disease-enhancer

In experimental VL, *L. donovani*-infected BALB/c mice have increased levels of IL-21 compared to uninfected controls suggesting a role for IL-21 in susceptibility [36]. In line with this, evidence suggests that IL-21/IL-21R signalling regulates antibody isotype switching especially IgG subclasses, IgG1 and IgA [37], the former associated with progression of leishmaniasis [35]. Conversely in CL, signalling via IL-21 appears to be dispensable to the infectious response by *L. major* as C57BL/6 IL-21R-deficient mice displayed similar disease progression to WT C57BL/6 mice despite increased Th1 responses [38]. Altogether, the contribution of IL-21/IL-21R to leishmaniasis remains a conundrum with limited studies on gene-deficient and receptor-deficient experiments to validate a distinct role. However, the combined reports do tend to emphasize a disease-enhancing role, particularly in VL.

### 3.4 IL-4 and IL-13 are protective in VL but pathogenic in species-specific CL

IL-13 and IL-4 are Th2 cytokines that share the common IL-4 receptor alpha (IL-4Rα) subunit for signalling [6]. This receptor interacts with the gamma common (γc) chain to form the type 1 IL-4 receptor, signalling IL-4, or with IL-13 receptor alpha 1 (IL-13Rα1) chain to form the type 2 IL-4/IL-13 receptor [6], signalling IL-4 and IL-13. IL-4 and IL-13 are implicated to play a major paradoxical role in *Leishmania* infections in mice.

IL-4/IL-13 and IL-4Rα-deficient BALB/c mice are protected from *L. major* but IL-4/IL-13 is host-protective in *L. donovani*-induced VL [39], [40]. However, IL-4Rα signalling appears to depend on the *Leishmania* species/sub-species initiating infection as *L. major* infection was also unchanged in IL-4/IL-4Rα-deficient mice [41]. Moreover, pleiotropic effects of IL-4 and IL-13 influences cell-specific roles for IL-4Rα signalling to *Leishmania*. Particularly, BALB/c mice deficient of IL-4Rα on CD4^+^ T cells are resistant to *L. major* infection [42] but not to *L. mexicana* infection [43], further identifying species-specific differences. These differences could be explained by the Th2 vs Th1-promoting role of IL-4 and IL-13. Traditionally, IL-4 and IL-13 are associated with Th2-dependent susceptibility to intracellular infection [5], [44]. However, this central dogma has been challenged extensively after reports demonstrated that IL-4 can instruct Th1 anti-leishmanial responses via induction of IL-12 by DCs and reduced IL-10, so-called “IL-4 instruction theory” [45]. In support of this, both *L. major* susceptible BALB/c and resistant C57BL/6 mice produce IL-4 in the early phase of infection, however this is sustained in susceptible mice but redirected in resistant mice by IL-12-dependent mechanisms [46]. In line with this, IL-4Rα deficiency on DCs in BALB/c mice rendered animals hypersusceptible to *L. major* infection [44] and was unable to confer resistance in a DC-mediated vaccination approach [47]. These two studies suggested that biological quantities of IL-4 acting on DCs *in vivo* is host-protective in mice, contrary to observations during *L. mexicana* infection where the disease is unaltered (*manuscript in preparation*), again highlighting species-specific requirements of IL-4 in Th1 vs Th2. Interestingly, IL-12-mediated DC instruction is confined to IL-4 in leishmaniasis so far, as IL-4 treatment, but not IL-13, enhanced DC-derived IL-12 production [39]. Altogether, the Th1 priming role of IL-4 is evident in VL but the cell-type specificity for protective effects of IL-4/IL-13 are still under investigation. Clearly, a protective effect on macrophages/neutrophils [48] and T cells have been ruled out [49]. Overall, IL-4 and IL-13 are pathogenic in CL but protective in VL although cell-specific effects of IL-4 and IL-13 appear to differentially regulate this response.

### 3.5 IL-9 is pathogenic in CL and unexplored in VL

IL-9, produced mainly by Th2 cells, is reported to drive susceptibility in *Leishmania* infection. For instance, high expression of IL-9 has been exhibited in BALB/c mice infected with *L. major* and neutralization of IL-9 protects mice by inducing protective Th1 responses and enhanced macrophage microbicidal effector functions [50]. Overall, studies on IL-9 in CL are limited and no studies on the role of IL-9 in VL, an aspect that can be explored further.

## 4 IFN-γ is anti-leishmanial but the magnitude differs in *Leishmania spp*.

The interferon (IFN) family consists of type I (IFN-β, IFN-κ, IFN-ɛ, IFN-ο, IFN-τ and IFN-δ), type II (IFN-γ) and type III (IFN-λ). In leishmaniasis, IFN-γ is the most well-studied. The expression of this cytokine activates STAT-1 to induce T-bet, the master transcription factor regulating Th1 cells [5]. Indeed, IFN-γ is the signature cytokine for mediating Th1/Type I responses while concomitantly inhibiting the activity of Th2 cells [5]. In intracellular pathogens, IFN-γ upregulates antigen processing and presentation pathways and enhances the release of oxygen radicals for parasite killing [51]. Moreover, it inhibits the activity of immunosuppressive IL-10 but induces the activity of other pro-inflammatory cytokines (IL-1, IL-6 and TNF-α). Besides T-cell activity, IFN-γ modulates B-cell activity by stimulating IgG2a class switching in B-cells [52]. Altogether, these multi-faceted contributions to Th1/Type 1 make IFN-γ a vital endogenous modulator for *Leishmania* clearance in the host.

An early study highlighted that *L*. *major*-infected IFN-γR^−/−^ or IFN-γ^−/−^ C57Bl/6 mice developed fulminant CL accompanied by enhanced Th2 responses [53]. However, its magnitude differs amongst *Leishmania* strains. For instance, the levels of IFN-γ secreted during *L*. *mexicana* infection are noticeably inferior compared to levels secreted during *L*. *major* infection [54]. Moreover, IFN-γ appears to be dispensable for early control of *L. amazonensis* infection in IFN-γ^-/-^ C57BL/6 mice but critical for control in the later stages of infection [55] and reflect a dependence on IFN-γ as parasite burden increases. In parallel, STAT1^-/-^ C57BL/6 mice resolve *L. major*-induced footpad lesions [56] and T-bet^-/-^ mice are significantly susceptible to *L. major* with a Th2 phenotype [57]. Altogether, this highlights a synergism of IFN-γ, STAT 1 and T-bet in the anti-leishmanial response.

In VL, IFN-γ^-/-^ BALB/c display increased parasite burden and delayed granuloma maturation at week 2 post*-*infection, although surprisingly by week 8, one-fifth of the knockout mice presented fully developed granulomas [58]. Thus, endogenous IFN-γ is essential for early granuloma development during VL infection and later may be compensated by the accumulation of other Th1/Type 1-enhancing factors. IFN-γ has also been evaluated in drug therapy. Accordingly, IFN-γ^-/-^ C57BL/6 mice showed no response to sodium stibogluconate treatment at low doses (100 mg/kg) but parasite replication was inhibited at a higher dose indicating a dose-dependent effect in therapy. In contrast, these mice were fully responsive to amphotericin B (AmB) treatment [59], confirming that not all anti-leishmanial drugs are dependent on IFN-γ. Collectively, despite differential responses during the early stages of infection, IFN-γ ultimately confers protection to all forms of leishmaniasis.

## 5 IL-6 cytokine family is an unsolved paradox

The IL-6 family consists of interleukins (IL-6, IL-11) and others such leukemia inhibitory factor and closely related oncostatin M [60], which signal via the gp130 receptor. Together, they influence pro-inflammatory/anti-inflammatory responses, B-cell stimulation and balanced regulation of regulatory and effector T cells [60]. In this family, IL-6 is confirmed to play a role in leishmaniasis although the nature of this role is controversial.

IL-6 is released by DCs, activated T-lymphocytes, monocytes, fibroblasts and activated macrophages via both autocrine and paracrine signaling. IL-6 is one of the greatest oxymora in leishmaniasis because multiple studies have reported discordant results and it has been suggested to promote, suppress or effect no change in host defense to *Leishmania*. For instance, deletion of endogenous IL-6 in C57BL/6 mice enhanced control of *L. donovani* replication together with increased levels of circulating IFN-γ [61]. Concomitantly, the absence of IL-6 receptor signaling during hepatic *L. donovani* infection favored Th1-type responses and parasite killing at the expense of severe liver pathology [62]. Altogether, this highlights an immunopathological role for IL-6 during infection, possibly exacerbated because it inhibits IFN-γ-mediated gene expression and macrophage activation.

Conversely, a host-protective role for IL-6 in DC T-cell priming was reported as the efficacy of DC therapy depended on BMDC-derived IL-6 to suppress the expansion of IL-10^-^producing T-cells during *L*. *donovani* infection [63]. Similarly, IL-6 is associated with host-protection in CL as epidermal IL-6 expression was vital to the induction of Th1 immunity as demonstrated by the non-healing phenotype in C57BL/6 IL-6^-/-^ [64]. Contrastingly, C57BL/6 IL-6^-/-^ control *L. major* efficiently as littermates [65]. This was confirmed later where there were no differences in the progression of disease in BALB/c IL-6^-/-^ and WT mice, however, IL-6^-/-^ had lower levels Th2 and Th1 populations [66]. Overall, endogenous IL-6 plays a pleiotropic role in leishmaniasis and pinpointing a disease-defining role has proven challenging, perhaps due to the shared gp130 receptor and counter-regulation of inflammatory responses.

## 6 TNF cytokine family are protective to VL but strain-specific in CL infection

The TNF cytokine family includes 19 ligands and 29 receptors with TNF-α and lymphotoxin-α (LT-α) being the most studied. TNF is crucial in the modulation of immunological responses by activating NF-κβ and pro-inflammatory responses [67], involved in the activation and proliferation of naïve and effector T-cells or can either suppress or expand Tregs. Thus, it is not surprising that TNF is important in regulating immune responses during *Leishmania* infection.

TNF-α is required for the expansion of IFNγ^+^CD4^+^ T cells that promote protection in VL. In support, B6.TNFα^−/−^ or B6.LTα^−/−^ mice are susceptible to hepatic *L. donovani* infection characterized by defective granuloma assembly, reduced CD4^+^ Th1 cytokines and impaired iNOS [68]. In parallel, B6.TNF^-/-^ mice in CL yielded similar yet unexpected results. Notably, TNF-α-deficient C57BL/6 mice were highly susceptible to *L. major* BNI strain despite increased levels of systemic IFN-γ [69] and iNOS yet B6.TNF^-/-^ mice were partially resistant to *L. major* Friedlin strain highlighting that the role of TNF-α during *L. major* infection is sub-strain-specific ranging between protection and susceptibility [70]. On the latter, TNF type 1 receptor (TNFR1) is implicated in susceptibility in experimental mice. Notably, TNFR1^-/-^ mice controlled *L. major* infection through IFN-γ and iNOS production [71]. While the role of other family members has not been explored in leishmaniasis, LTα/LTβ (TNF-β and TNF-γ, respectively) has been explored by its interaction to form membrane-bound LTα_1_β_2_. The absence of membrane lymphotoxin via deletion of LT-β confers resistance to *L. major* infection via a strong Th1 immune response [72]. Together, these studies suggest that TNF-α is indispensable in the control of VL but the unsolved paradox is its opposing effects on strain-dependent CL infections, suggesting that the effects of TNF-α may proceed independently of Th1-dependent immunity or ROS as seen in other studies. These independent effects, and the function of other members in the TNF superfamily, remain to be conclusively examined in leishmaniasis.

## 7 The interleukin-12 family

The IL-12 family are heterodimeric cytokines with an α-chain subunit (P19, P28, P35) paired with β-chain subunit [P40 or Epstein Barr virus-induced gene 3 (EBI3)] and include IL-12, IL-23, IL-27, IL-35 and IL-39 [60]. IL-12 signals via IL-12Rβ1 and IL-12Rβ2, IL-23 signals via IL-12Rβ1 and IL-23R whereas IL-27 and IL-35 use gp130 with WSX-1 and IL-12Rβ2, respectively [60]. Despite these cytokines sharing molecular partners, they display several distinct features.

### 7.1 IL-12 is host-protective to induce IFN-γ

IL-12 is a potent inducer of IFN-γ that is required for successful control of *Leishmania* infection [48]. However, early production of IL-12 does not guarantee resistance to *Leishmania* but rather appears to influence the ability of naïve CD4^+^ T cells to express the IL-12Rβ2 chain required for IL-12-mediated IFN-γ production [73]. Neutralization of IL-12 exacerbates *L. donovani* [74]. Clinically, IFN-γ production and cytotoxic activity of NK cells are enhanced by exogenous coculture of IL-12 with lymphocyte cultures from VL patients [22]. Similarly, IL-12-deficient mice are susceptible to *L. major* infection but IL-12 is not required for early control of *L. mexicana* infections [54]. Given the requirement for IL-12 in disease control, it is not surprising that *Leishmania* parasites selectively modulate IL-12 production by DCs for IL-10 to promote their survival [75]. Importantly, IL-4Rα-deficient DCs have reduced IL-12 production in *L. major* infection but increased IL-10 and IL-23 rendering the animals hypersusceptible to disease [39]. This highlights IL-4 as an instructor of DC-derived IL-12 for disease control whilst supporting a role for IL-10 and IL-23 in disease progression. Notably, IL-4 instruction of IL-12 may be cell-specific since IL-4Rα-deficient B cells have elevated levels of IL-12 that promote host protective Th1 response in *L. major* infection [6].

### 7.2 IL-23 may be disease-promoting but gene-deficient studies are lacking

In a clinical study, IL-23 was shown to correlate with healed lesions in CL due to *L. major* compared to non-healing patients [76], highlighting its role in limiting immunopathology. However, murine studies imply a disease-promoting role for IL-23 which is surprising considering that it shares the P40 receptor with IL-12, which is host-protective. For instance, BALB/c mice infected with *L. major* show increased levels of IL-23 in LNs [77]. This is accompanied by increased IL-17 and increased neutrophil recruitment into lesions, the latter known to enhance disease development [78]. IL-17 also contributes to susceptibility to CL caused by *L. major* [77], suggesting an additive effect of IL-23 to induce IL-17 for disease progression. Alternative activated macrophages (M2), which support growth and replication of *Leishmania*, were shown to secrete high amounts of IL-23 in autoimmune disease [79] though a role for IL-23 in M2 activation in *Leishmania* remains to be elucidated. Taken together, these inference studies tend to imply IL-23 could be disease-promoting but IL-23-deficient mice remain to be tested in any form of leishmaniasis.

### 7.3 IL-27 is an unsolved paradox

IL-27 is a regulatory cytokine in leishmaniasis due to its ability to exert pleiotropic effects on Th1, Th2 and Th17 functions. Its suppressive activity is linked to IL-10 secretion from activated CD4^+^ T cells via autocrine action of IL-21. Moreover, IL-27 is an early inducer of T-bet and Th1 differentiation in the absence of IL-12 and independent of STAT4 and IFN-γ but dependent on STAT1 [5]. With these many roles, its contribution to leishmaniasis is also diverse.

IL-27 was found to correlate with healed *L. major* lesions, similar to IL-23 [76]. Whilst IL-27 was protective to *L. major* [76], IL-27 enhanced disease during *L. amazonensis* infection, an effect mediated by IL-10 [80]. Comparatively, IL-27R-deficient mice (WSX-1^–/–^) show more severe lesions to *L. major* [81] due to a delayed Th1 response, highlighting its role in immunopathology. Controversially, another study demonstrated that WSX-1^-/-^ mice resolved lesions [82] suggesting that IL-27 is dispensable to the infectious process. These discordant reports could be explained by reciprocal regulation of IL-27 on IL-10 and Th17. IL-27 induces CD4^+^ T cell-derived IL-10, which is canonical disease-enhancer in leishmaniasis, whilst at the same time it suppresses Th17/IL-17 [82], both of which can promote disease progression when present.

In a murine model of VL, IL-27R-deficient C57BL/6 mice displayed a significant reduction in liver parasites but developed severe liver pathology [62]. This suggests that while IL-27 is involved in VL susceptibility, it may limit the severity of hepatic immunopathology [62] hence inhibiting IL-27 could be targeted for VL immunotherapy. However, in terms of CL, caution should be noted as the bulk of the literature tends to suggest that IL-27 is an unsolved paradox, a disease-enhancer and a host-protector depending on the parasite species initiating infection.

## 8 IL-10 cytokine family

### 8.1 IL-10 is a potent factor for exacerbating VL but a paradox in species-specific CL

The IL-10 family of cytokines includes IL-10, IL-19, IL-20, IL-22, IL-24 and IL-26. Although initially thought to be produced by Th2 cells that blocks the synthesis of Th1 cells, IL-10 can also be produced by macrophages, DCs, B-cells, Tregs and Th17 cells [60], [83]. Additionally, IL-10 directly blocks macrophage and DC-derived IL-12 thus impairing proliferation of Th1 cells and IFN-γ development [84]. This inhibitory role of IL-10 is exemplified during leishmaniasis.

Although early work suggested that IL-10 is not involved in Th1 differentiation due to anti-IL-10 treatment of C3H/HeN and BALB/c mice [85], later studies contradicted this conclusion as *L*. *major* infected IL-10^-/-^ BALB/c mice footpad displayed 1000-fold lesser parasite at five weeks post-infection compared to WT BALB/c [86]. This highlights the importance of gene-deficient studies in comparison to antibody-based inhibitors to resolve cytokine-dependent effects *in vivo*. Paradoxically, in *L*. *mexicana* and *L*. *amazonensis*, IL-10 had little effect on lesion outcome as infected IL-10^-/-^ BALB/c failed to control infection [87]. Later, Buxbaum and Scott [88] revealed that C57BL/6 IL-10^-/-^ mice cured *L*. *mexicana* lesions but could not resolve lesions caused by *L*. *amazonensis* hence IL-10 seems not to play a vital role in determining the phenotype that ensues in *L*. *amazonensis* infection [89]. These observations could be reconciled by the finding that in chronic CL infection, despite expression of IFN-γ, disease progresses perhaps due to IL-10^+^ Th1 CD4^+^ cells that suppress IFN-γ activity [90].

While CL reports indicate a species-specific role, this cytokine strongly promotes visceral infection as IL-10-deficient mice showed enhanced parasite clearance [91]. Interestingly, when IL-10^-/-^ mice were treated with αIL-12, their parasite burden was comparable to WT BALB/c control, highlighting counter-regulatory roles for IL-10 and IL-12 [92]. Chronic VL by *L. donovani* is linked to CD4^+^ T-cells expressing both IL-10 and IFN-γ [83], which may reflect IL-10 suppression or antagonism of IFN-γ, as documented in CL. Overall, IL-10 is an important immune-deactivating cytokine in VL and its inhibition could prove very promising in the immuno-therapeutic discovery platforms however its effects must be delineated at a species-specific level for CL.

### 8.2 IL-22 confers protection in CL

IL-22 is mainly produced by CD4 T-cells and NK cells. Functionally, it is presumed to modulate epithelial innate immunity [60] but also crucial in chronic inflammatory disorders and infectious diseases. Moreover, IL-22 plays a paramount role in tissue repair, a function complemented by its anti-microbial properties [93]. Evidence for this has been reported in leishmaniasis. For example, IL-22 limits *L. major* pathology as IL-22^-/-^ C57BL/6 mice display enhanced ear swelling than WT [94]. In MCL murine model, a similar trend was observed as *L. braziliensis* infected IL-22^-/-^ had significantly larger lesions than WT at the site of infection [94]. Notably, IL-22 only acted at high doses of parasites suggesting that IL-22 might act only when inflammation reaches a threshold [94].

These results were contradicted in another study as disease response was unchanged in *L. major* infected IL-22^-/-^ C57BL/6 mice compared to WT [95]. There is little about the role of IL-22 in experimental VL, however, this cytokine protects hepatocytes [60] suggesting a role in regulating hepatomegaly, a hallmark of VL. Overall, IL-22 would make a promising candidate for CL and MCL-induced leishmaniasis due to the need for wound healing. Further studies are necessary to delineate its role in VL.

## 9 IL-17 cytokine family

### 9.1 IL-17 is an unsolved paradox in leishmaniasis

IL-17A is the founding member of this family, together with IL-17B, C, D, E and F, and the most well-studied in leishmaniasis. IL-17 increases the pathology of *L. major* infection in BALB/c mice in an IFN-γ rich environment [96] via neutrophil recruitment. Similarly, IL-17 also appears to enhance muscosal leishmaniasis [97] and VL caused by *L*. *donovani* [98]. Contrastingly, Th17 and IL-17 play a role in host protection against VL, complementary to IFN-γ and Th1 subset. In support, in *L. infantum*-infected C57BL/6 mice, IL-17A synergizes with IFN-γ to control infection through NO [99]. Similarly, IL-17RA^-/-^ mice are susceptible to *L. infantum* infection with reduced IFN-γ-expressing CD4^+^ T cells [99]. This could be due to IL-17 stimulation of macrophage-derived IL-1, TNF-α, and NOS. Overall, the precise role of IL-17 in leishmaniasis remains an unsolved paradox and more studies are warranted to consolidate its function in CL and VL.

## 10 Transforming growth factor beta (TGF-β) family is a disease-enhancer

TGF-β family includes three isoforms; TGF-β1, TGF-β2 and TGF-β3 with pleiotropic and redundant functions that control proliferation, differentiation and immunosuppression of cells in all tissues [100]. In leishmaniasis, TGF-β1 is the most studied and enhances arginase for polyamine production that favors parasite survival. In support, neutralization of TGF‐β protects BALB/c mice against *L. donovani* infection and TGF-β complementary to IL-10 positively correlates with the disease in humans [101]. Similarly, TGF‐β has been implicated in disease progression in experimental CL caused by *L*. *major* [102], altogether highlighting its role as a disease-enhancer in CL and VL.

## 11 Concluding remarks

Cytokine-based immunotherapy, or co-administration with drugs as an immunochemotherapeutic approach, is a promising tool for mitigating drug resistance and alleviating drug toxicity in leishmaniasis treatment. However, central to designing, developing and implementing these approaches is a solid understating of cytokine interplay during disease. Notably, the IFN family (IFN-γ) and IL-12 are unambiguous as host-protectors in both CL and VL. However, implementing cytokine-based immunotherapeutics for CL would be delicate and challenging as more cytokines are unsolved paradoxes in CL than VL, some of which are based on species specificity or appear dispensable to the response altogether.

This raises a pertinent question, as to why infection with CL-causing species, but not VL-causing species, is associated with pronounced paradoxical functions of many cytokines? A very simplistic reason for this could be due to the fact that cytokine and cytokine receptor deficiencies are more widely studied in experimental CL than in experimental VL. For instance, little to no data are available on the contribution of cytokines such as IL-23, IL-22, IL-9, IL-7, IL-15 and IL-17A, nor their receptors, to experimental VL. It is possible that investigations into these less-explored cytokines could reveal immunoregulatory or, paradoxical roles during VL. Furthermore, the redundant, synergistic and pleiotropic action of cytokines, due to the sharing of cytokine receptors and hierarchy of action, could account for different cytokine responses during pathogenic challenges [5]. Other contributing factors are discussed below, which may involve the experimental model used, host and parasite genetics and/or immune cells and cytokine milieu during infection.

Majority of the studies reported herein used susceptible BALB/c mice as the experimental model for genetic deletion of cytokines and cytokine receptors. However, experimental VL in BALB/c mice is self-limiting due to the development of hepatic granulomas [103]. In contrast, granuloma formation is uncharacteristic with CL [40] and disease in BALB/c is progressive and even fatal. Thus, it is tempting to speculate that the inherent self-healing pathway in BALB/c mice to VL is perhaps less amendable to cytokine modulation than observed in experimental CL and could have resulted in the discrepancy in cytokine paradoxes between both diseases. Notably, parasite and host genetics regulating visceralization and virulence of VL-causing species are clearly distinct from those regulating these mechanisms in CL-causing species [104]. For instance, the *A2* gene locus has been demonstrated to be important for visceralization of VL caused by *L. donovani* and *L. infantum*, but in *L. major* and *L. tropica* causing CL, *A2* is a pseudogene [105], [106]. Similarly, natural resistance-associated macrophage protein 1/Lsh (NRAMP/Lsh) gene located in chromosome 1, a cation transporter, controls susceptibility to *L. donovani* but not to *L. major* [107]. Hence, differences in visceralization and virulence genes during infection may explain why acquired cytokine responses differ significantly in the host.

Immune cell activation also differs during CL and VL. Particularly, *L*. *donovani* prefers to infect tissue-resident macrophages such as those in the spleen, bone marrow and liver (Kupffer cells) [108], whereas, *Leishmania* spp causing CL are preferentially taken up by monocytes, macrophages and inflammatory DCs in draining lymph nodes [109]. As the quality and quantity of cytokine action in these different host cell populations differ, to induce varying immune responses, this may account for the functional cytokine discrepancy during pathogenic challenge [103]. Lastly, the activation and differentiation of Th populations in either pathogenic challenge is not synonymous. For instance, the Th1/Th2 dichotomy has strongly been linked to the outcome CL in mice but this is not the case in VL, where both IFN-γ (Th1) and IL-4 (Th2) are needed for host-protection. Moreover, recent studies have highlighted that other Th cell populations, such as T regulatory cells and Th17 cells, may play a key role in modulating disease outcome (Fig. 1). As with macrophages, these diverse Th populations differ in their capacity to secrete and respond to cytokines, which may further account for the different cytokine responses seen upon pathogenic challenge during cutaneous and visceral disease.

Thus, more studies on cytokine interplay in clinical and experimental systems are necessary to alleviate the challenges faced by the available leishmaniacides if combinational immunochemotherapeutic approaches, involving cytokines and their receptors, are to be successful.

## CRediT authorship contribution statement

**Bernard Ong’ondo Osero:** Conceptualization, Writing - original draft, Data curation, Writing - review & editing. **Raphael Taiwo Aruleba:** Conceptualization, Writing - original draft, Data curation, Writing - review & editing. **Frank Brombacher:** Writing - review & editing, Supervision, Funding acquisition. **Ramona Hurdayal:** Conceptualization, Writing - original draft, Writing - review & editing, Supervision, Funding acquisition.

## Declaration of Competing Interest

The authors declare that they have no known competing financial interests or personal relationships that could have appeared to influence the work reported in this paper.
